# Thyrotoxicosis: Unraveling the Mystery of Fever

**DOI:** 10.7759/cureus.85747

**Published:** 2025-06-11

**Authors:** Zeinab K Majed, Miril Janji, Hadil Basma, Paola Atallah

**Affiliations:** 1 Department of Endocrinology, Diabetes and Metabolism, Faculty of Medicine, Lebanese University, Beirut, LBN; 2 Department of Endocrinology and Metabolism, Saint George Hospital University Medical Center, Beirut, LBN

**Keywords:** fever, fever of unknown origin, hyperthyroidism, tachycardia, thyroiditis, thyrotoxicosis

## Abstract

Subacute thyroiditis (SAT) is a transient inflammatory thyroid disorder, typically following an upper respiratory tract viral infection, and can rarely present as fever of unknown origin (FUO). Considering endocrine diseases in the differential diagnosis of fever is essential to prevent delayed diagnosis and unnecessary investigations.

We report a case of a 65-year-old male who presented with a prolonged fever of two to three weeks, unresponsive to antibiotics, starting with mild upper respiratory symptoms. Extensive infectious and other workups were negative. Thyroid function tests revealed suppressed thyroid-stimulating hormone (TSH) and elevated free thyroxine (T4) and triiodothyronine (T3) levels. Thyroid ultrasound findings were consistent with thyroiditis. The patient was diagnosed with SAT and treated with corticosteroids, leading to rapid symptom resolution.

SAT can be overlooked in the evaluation of FUO, particularly in the absence of classic symptoms. Thyroid function testing can be considered in the routine workup of FUO. Treatment should be based on symptoms, with an appropriate corticosteroid regimen if needed, and proper follow-up is essential to monitor thyroid recovery and potential progression.

## Introduction

All conditions with elevated levels of thyroid hormones are called thyrotoxicosis, regardless of the underlying mechanism [1]. It amplifies catecholamine signaling, leading mainly to adrenergic symptoms (e.g., palpitations, heat intolerance, tremor, and increased bowel movements) [2]. Other manifestations can include hypermetabolism and neuromuscular symptoms (e.g., proximal muscle weakness) [3]. If left untreated, it can cause cardiac arrhythmias, congestive heart failure (CHF), osteoporosis, infertility, oligomenorrhea, uncontrolled diabetes mellitus (DM), and reduced exercise tolerance [1].

Graves’ disease is the most common cause of hyperthyroidism (70%), followed by thyroid multinodular goiter (16%). It can be caused by other uncommon conditions, such as subacute thyroiditis (SAT) (3%) and drug-induced cases (9%) [4].

Thyroiditis can be caused by painful conditions, such as SAT and suppurative thyroiditis, as well as by painless conditions, such as drug-induced thyroiditis, postpartum thyroiditis, and Riedel thyroiditis [5].

SAT generally follows a viral upper respiratory infection and causes the further release of preformed thyroid hormone due to inflammatory destruction of thyroid follicles [6,7]. It is considered the most common cause of painful thyroiditis, with females more likely to be affected, and the age at presentation ranging from three to 78 years old (average ± SD: 35.2 ± 11.2 years) [6,8]. Painless SAT is rare and can come as a fever of unknown origin (FUO) [9].

In 1961, FUO was originally defined by Petersdorf and Beeson as a body temperature above 38.3°C (101°F) on three or more occasions for at least three weeks’ duration, if undiagnosed after preliminary investigations [10]. The causes can vary. Overall, infections have been the most common (37.8%), followed by non-infectious inflammatory diseases (20.9%) such as vasculitis, granulomatous disorders, and malignancies. Other diseases (6.5%) are categorized as miscellaneous causes, which comprise SAT. The diagnosis remained irresolute in 23.2% of cases [11,12].

Endocrine disorders are a very infrequent cause of FUO [13]. Since 1961, FUO has remained a challenging diagnosis, with the most wide-ranging differential diagnosis.

## Case presentation

We report a case of a 65-year-old male, a non-smoker, with a medical history of benign prostatic hyperplasia managed with tamsulosin, and dyslipidemia treated with rosuvastatin 10 mg. On November 22, 2024, he was admitted to our hospital for an unresolved fever lasting 18 days, which did not respond to a course of amoxicillin/clavulanic acid prescribed by his primary care physician for a suspected upper respiratory tract infection. The fever was accompanied by a mild headache and a five-day history of a new-onset dry cough. Additionally, he reported increased palpitations and eye dryness and redness, for which he had been using artificial tears.

He denied experiencing gastrointestinal, genitourinary, neurological, or musculoskeletal symptoms, as well as periorbital pain, jaw claudication, neck rigidity or pain, night sweats, or skin rashes. There was no history of consuming unpasteurized milk, contact with animals, recent surgical or dental procedures, new medications, or weight loss. As a result, he was hospitalized for evaluation and management of fever.

Upon admission, his vital signs were all within normal ranges except for his temperature of 39°C and heart rate of 110/min. A physical examination was unremarkable. Blood laboratory tests were significant for mild leukocytosis (Table 1) and increased inflammatory markers (Table 2).

**Table 1:** Complete blood count.

Laboratory test	Value	Units	Reference range
White blood cells	11.64	10^9/L	4-10
Hemoglobin	12.4	g/dl	13-17
Hematocrit	37	%	40-50
Platelets	264	10^9/L	150-450

**Table 2:** Trends of inflammatory markers.

Laboratory test	Reference range	October 22, 2024	November 22, 2024	November 24, 2024	November 26, 2024	November 28, 2024	November 31, 2024
Erythrocyte sedimentation rate	0-15 mm/hour	-	90	92	-	-	-
C-reactive protein	<0.6 mg/dl	17.38	23.78	17.79	24.89	23.72	22.91

Other laboratory tests, including kidney function, liver function, electrolytes, uric acid, antinuclear antibody (ANA), and procalcitonin, were all within normal range. An extensive workup for FUO was performed, including microbiology studies such as panculture, *Cytomegalovirus* (CMV) IgM, Epstein-Barr virus (EBV), purified protein derivative (PPD), *Brucella* antibodies, and *Salmonella*, all of which were negative.

Additionally, a cardiac ultrasound and a chest-abdomen-pelvis CT scan revealed no evidence of endocarditis or infections, respectively.

**Figure 1:**
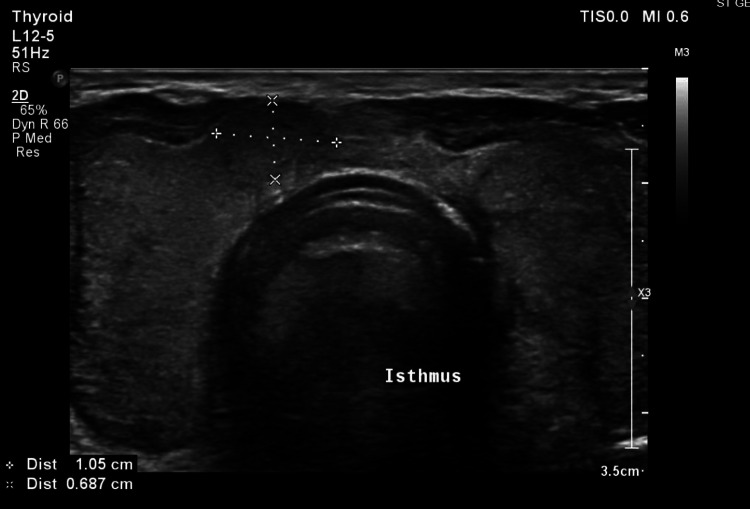
Ultrasound showing the enlarged heterogeneous thyroid with right lobo-isthmic nodule.

**Figure 2:**
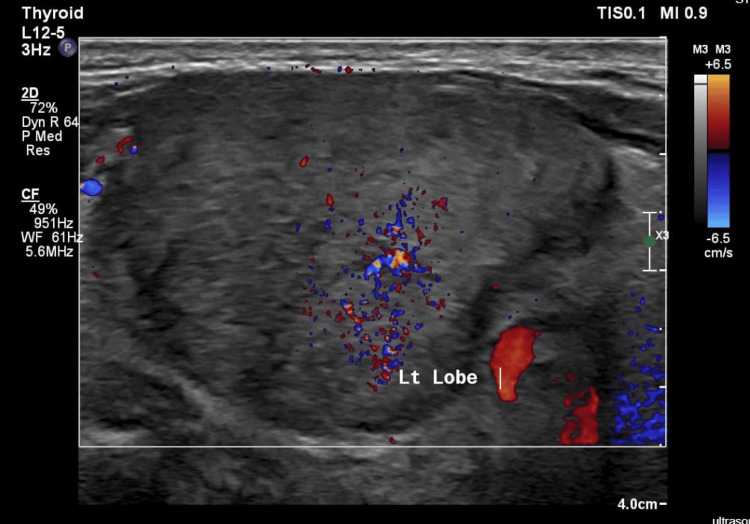
Ultrasound showing the enlarged heterogeneous thyroid with minimal increased vascularity.

**Figure 3:**
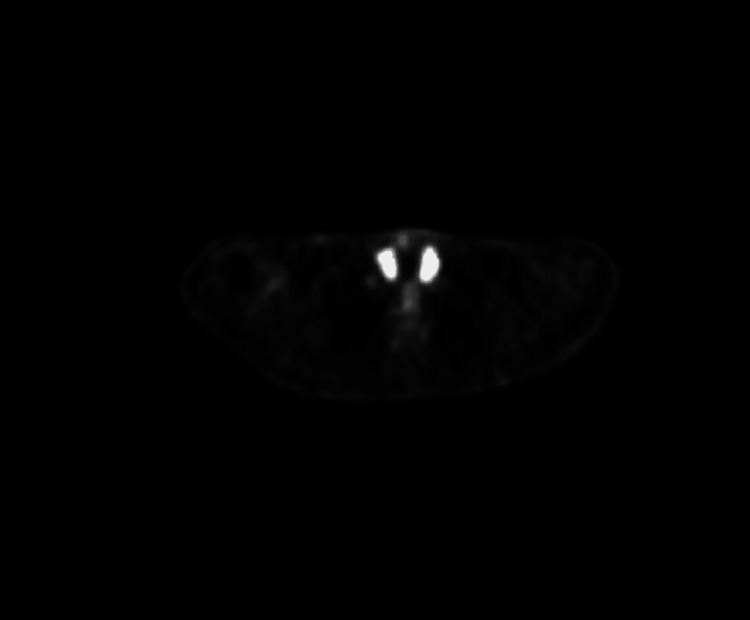
PET scan showing diffuse increased fluorodeoxyglucose uptake within the enlarged thyroid gland.

Due to the persistence of fever without a clear source of infection and without improvement on continuous intravenous ketoprofen, a PET scan was ordered, and as part of the evaluation for tachycardia, thyroid function tests were conducted on November 24, 2024. The results revealed low thyroid-stimulating hormone (TSH) levels and elevated free thyroxine (T4) and triiodothyronine (T3) levels (Table 3). A thyroid ultrasound done on November 28, 2024, showed an enlarged heterogeneous echotexture of the thyroid gland isthmus indicating post-thyroiditis, with minimal increase in vascularity on echo Doppler, and a 1 cm nodule in the right lobo-isthmic area classified as TIRADS (Thyroid Imaging Reporting and Data System) III, and no cervical lymph nodes (Figures 1, 2). A PET scan done on November 28, 2024, after doing the thyroid ultrasound, showed diffuse increased fluorodeoxyglucose (FDG) uptake within the enlarged thyroid gland, which could be related to thyroiditis (Figure 3).

**Table 3:** Initial and subsequent thyroid function tests.

Laboratory test	November 24, 2024	December 2, 2024	January 17, 2025	Units	Reference range
Thyroid-stimulating hormone (TSH)	0.06	4.66	5.95	microU/ml	0.27-4.2
Free T4	3.99	8.6	-	ng/dl	0.92-1.68
Free T3	5.97	2.71	-	pg/ml	2.5-4.3
TSH receptor antibodies (TRAb)	-	0.63	-	IU/L	<0.8
Anti-thyroperoxidase (anti-TPO)	-	1.02	-	IU/ml	<34
Anti-thyroglobulin	-	0.61	-	IU/ml	<116

TRAb, anti-TPO, and anti-thyroglobulin antibodies were negative (Table 3), making the diagnosis of autoimmune thyroid diseases unlikely, so he was diagnosed with subacute thyrotoxicosis and started on propranolol 20 mg twice per day and slow tapering oral steroids (prednisolone). The patient’s fever and palpitations resolved soon after starting treatment, with follow-up thyroid function tests revealing normalization, then increased TSH, and he was clinically euthyroid (Table 3). He showed a good clinical evolution, assuring the diagnosis of SAT.

## Discussion

The diagnosis of SAT is mainly clinical, where neck/jaw pain - easily dismissed as dental pain - may be a clue to SAT [11]. Symptoms vary, with palpitations and neck pain (unilateral or bilateral) being the most common presentations (62.5%), followed by weight loss (50%), goiter, anxiety, fatigue, and heat intolerance (37.5%). Diaphoresis and hoarseness occur in 25%, and only 12.5% of SAT subjects may present with tremors, fever, mood swings, insomnia, hair loss, and ear or face pain [8].

Inflammatory markers such as erythrocyte sedimentation rate (ESR) are high, and radioactive iodine (RAI) uptake is low [7,8,14]. Ultrasonography shows heterogeneous hypoechoic parenchyma and decreased vascularity [7].

It follows a triphasic pattern with a self-limiting course [6]. Around 50% of patients present in their first symptomatic phase of thyrotoxicosis, which lasts for three to six weeks, followed by painless hypothyroidism (developed in one-third of patients), which can last up to six months. Most return to euthyroidism within 12 months of onset, except for 5-15% of patients who develop permanent hypothyroidism [7].

The goal in treatment is to ameliorate the symptoms. For tachycardia, beta blockers are required. For mild symptoms, treatment is not needed. In the painful phase, patients can be treated with nonsteroidal anti-inflammatory drugs (NSAIDs; e.g., ibuprofen 1200-3200 mg per day in divided doses) or corticosteroids for severe symptoms or if NSAIDs are not effective after four days of intake (e.g., prednisone 15-40 mg per day for one to six weeks, followed by tapering of 5 mg every two weeks). If hypothyroidism occurs, levothyroxine is used if the patient is symptomatic or of reproductive age, and it is mostly transient [7].

In a systematic review of treatment protocols for SAT, steroid therapy was the most effective for moderate to severe SAT, with low initial doses (15 mg) preferred compared to higher doses (30-40 mg). The review showed that a short tapering period was associated with greater recurrence rates [15].

Stasiak et al. noted that previously painless SAT was simply undiagnosed and that cases are increasing over the years [16]. Despite changing the quantitative definition of FUO with a qualitative one requiring obligatory investigations - including a minimal diagnostic workup such as C-reactive protein, complete blood count, electrolytes, creatinine, total protein, protein electrophoresis, liver function tests, lactate dehydrogenase, rheumatoid factor, cultures, chest X-ray, abdominal ultrasound and many others - tests that should be included in the workup remain a matter of debate [11].

Given the possibility for SAT to be painless or dismissed, our case emphasizes the importance of considering SAT in the differential diagnosis of FUO and including thyroid function tests (TFTs) in routine workup to prevent the patient from being subjected to unnecessary diagnostic tests and treatments.

## Conclusions

Fever is mostly due to infectious or inflammatory causes, but many endocrine disorders (thyrotoxicosis, carcinoid syndrome, pheochromocytoma, adrenal crisis) can present as fever. SAT is a rare thyroid disorder and is often overlooked, especially if it is painless. It should be considered in the differential diagnosis of FUO. A good clinical approach can aid in the early resolution of the diagnostic dilemma of fever.
